# The Role of the Environment in Horizontal Gene Transfer

**DOI:** 10.1093/molbev/msac220

**Published:** 2022-10-13

**Authors:** Hande Acar Kirit, Jonathan P Bollback, Mato Lagator

**Affiliations:** Veterinary and Ecological Sciences, Institute of Infection, University of Liverpool, Liverpool, Merseyside, United Kingdom; Laboratories of Molecular Anthropology and Microbiome Research, University of Oklahoma, Norman, OK; Department of Anthropology, University of Oklahoma, Norman, OK; Veterinary and Ecological Sciences, Institute of Infection, University of Liverpool, Liverpool, Merseyside, United Kingdom; School of Biological Sciences, Faculty of Biology, Medicine and Health, University of Manchester, Manchester, United Kingdom

**Keywords:** horizontal gene transfer, evolutionary barriers, distribution of fitness effects, gene-by-environment interactions

## Abstract

Gene-by-environment interactions play a crucial role in horizontal gene transfer by affecting how the transferred genes alter host fitness. However, how the environment modulates the fitness effect of transferred genes has not been tested systematically in an experimental study. We adapted a high-throughput technique for obtaining very precise estimates of bacterial fitness, in order to measure the fitness effects of 44 orthologs transferred from *Salmonella* Typhimurium to *Escherichia coli* in six physiologically relevant environments. We found that the fitness effects of individual genes were highly dependent on the environment, while the distributions of fitness effects across genes were not, with all tested environments resulting in distributions of same shape and spread. Furthermore, the extent to which the fitness effects of a gene varied between environments depended on the average fitness effect of that gene across all environments, with nearly neutral and nearly lethal genes having more consistent fitness effects across all environments compared to deleterious genes. Put together, our results reveal the unpredictable nature of how environmental conditions impact the fitness effects of each individual gene. At the same time, distributions of fitness effects across environments exhibit consistent features, pointing to the generalizability of factors that shape horizontal gene transfer of orthologous genes.

## Introduction

Horizontal gene transfer (HGT), or the transfer of genetic material between species rather than the vertical transfer from parent to offspring, is an important source of novel genetic variation and adaptive traits. HGT can play a critical role in adaptation and organismal evolution by alleviating the disadvantages of asexual reproduction both in prokaryotes and eukaryotes (Doolittle 1999; Ochman et al. 2000; Koonin et al. 2001). For example, 10% of the active genes in microinvertebrate Bdelloid rotifers were acquired from other species through HGT, enabling them to survive in extreme conditions without sex for 80 million years (Boschetti et al. 2012). HGT is also a major source of genetic and phenotypic diversity in nature, such as the interkingdom transfer of fungal carotenoid biosynthesis genes to insects that led to the red–green polymorphisms in pea aphids (Moran and Jarvik 2010). In bacteria, HGT can drive rapid adaptation to novel stresses, contributing to the evolution of societally impactful traits like antibiotic resistance (Moyes et al. 2020; Carr et al. 2021).

The outcome of a horizontal transfer event—whether the gene is eliminated or fixed in the population—is determined largely by selection and its interplay with the genetic drift (Soucy et al. 2015). In other words, whether a HGT event is successful depends critically on how the transferred gene(s) alter the fitness of their new host.

Bioinformatics studies identified environmental conditions as a major factor determining the fitness effect of a transferred gene (Aminov 2011; Fuchsman et al. 2017). In fact, genes can be beneficial in one, neutral in another, and detrimental in a third environment—as exemplified by antibiotic resistance genes (Bjorkman et al. 1998; San Millan et al. 2018). Similarly, in the absence of a corresponding energy source, expression of metabolic enzymes is costly, creating conditions for selection of complex regulatory systems prevalent among all microbes (Pál et al. 2005; Donati et al. 2018). Because the fitness effects of transferred genes are environment-dependent, the impact of HGT on the genetic composition of populations depends on environmental composition and heterogeneity (Bell 1997). Therefore, it is not surprising that large differences between genomes of different species or even between individuals of the same microbial species are frequently observed (Segerman 2012). In spite of its importance, a systematic experimental investigation of the role of environment in the maintenance of horizontally transferred genes is missing.

To experimentally test how environment impacts the fitness effects of horizontally transferred genes, we transferred and expressed 44 gene orthologs from *Salmonella enterica* subsp. *enterica* serovar Typhimurium to *Escherichia coli* recipient cells. The 44 orthologs used in this study were randomly selected from the *Salmonella* genome, avoiding genes that are known to be related to mobile genetic elements. These genes were used previously by us to test for the factors that determine their fitness effects in a single environment (Acar Kirit et al. 2020). HGT can occur through many different modes and between distantly or closely related species, with each scenario introducing a different set of factors that shape the fitness effect of transferred genes. We adopted this design as we were interested in focusing on a specific stage in the process of horizontal transfer—the moment when an orthologous gene is successfully transferred and expressed in a closely related host. Our chosen design allowed us to address the questions of whether 1) environment determined the fitness effects of individual genes; 2) it was possible to predict gene-by-environment interactions; and 3) the shape of the distributions of fitness effects of all 44 horizontally transferred orthologous genes changed between environments.

## Results

As non-expressed genes are unlikely to have a strong effect on fitness (Lynch and Marinov 2015), we ensured that all 44 genes were expressed to the same level by inserting green fluorescent protein (GFP) under the inducible promoter P_LtetO-1_ (hybrid of tetracycline repressor region and lambda left promoter region) (Lutz and Bujard 1997) and measuring the fluorescence intensity of the cells with flow cytometry in each environment.

**Fig. 1.**
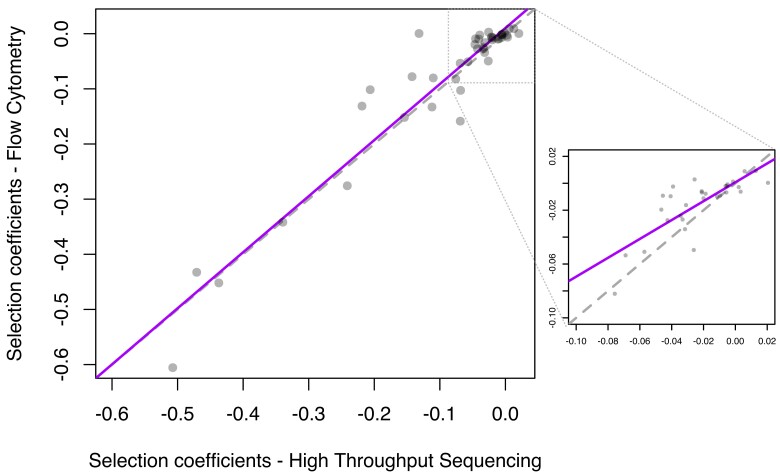
Comparison of the selection coefficients of transferred *Salmonella* orthologs measured in *Escherichia coli* with two different techniques: (y-axis) using individual competition experiments between each of the mutants and the wild type, measured using flow cytometry, and (x-axis) competing all mutants against each other at the same time and measuring the changes in their frequencies using HTS. Solid line is linear regression, F_1, 42_ = 461, *r*^2^ = 0.92, *P* < 0.001, with a slope of 1.017. Dotted gray line is x = y. The imbedded plot shows the correlation when only neutral and nearly neutral genes are considered (s > − 0.1) (F_1, 28_ = 45.68, *r*^2^ = 0.33, *P* < 0.001, slope 0.701).

We adapted a technique from (van Opijnen et al. 2009) to combine competition assays and high-throughput sequencing, enabling us to measure the fitness effects of all 44 transferred genes simultaneously in a single experiment. Using this technique, we estimated the relative fitness effect of each transferred gene on its new host *E. coli* in six physiologically relevant environments: two standard laboratory growth conditions (M9 and LB media), and four stress conditions that represent ecological conditions potentially experienced by *Salmonella* Typhimurium and *E. coli*—low oxygen (LO_2_), low pH (pH5) and antibiotics chloramphenicol (CAM), and trimethoprim (TMP). To determine the effects of the transferred genes we controlled for the effect of the environmental stress by competing each “mutant” strain carrying the transferred gene against the “wild type” in a pooled competition assay. Instead of the transferred gene, wild type carried a random DNA sequence that did not have a measurable effect on fitness (*s* < 0.001, Wilcoxon signed rank test, *P* = 0.24). Competition assays are traditionally performed through mixing of mutant and the wild type strains in 1:1 ratio at the beginning of the competition and the subsequent quantification of their relative frequencies at the end of competition, for each mutant strain individually; the selection coefficients (*s*) were then estimated from those frequencies (Elena et al. 1998). Previously, to obtain extremely precise estimates of fitness coefficients of each mutant strain (with precision of estimated selection coefficients *Δs* ≈ 0.005), we employed flow cytometry for the quantification of mutant and wild type frequencies, where the two were genetically labeled with different fluorescent markers (Acar Kirit et al. 2020). Here, we adopted a more efficient approach that lends itself to high throughput assays across multiple environments, without a loss in the precision of the selection coefficient estimates (*Δs*, effect size of flow cytometry measurements, *x̄* = 0.005, *σ* = 0.005, were not significantly different than those of high throughput sequencing (HTS), *x̄* = 0.007, *σ* = 0.005; *t*_57.83_ = 0.702, *P* = 0.486). Specifically, we performed pooled competition experiments and employed HTS to estimate relative frequencies of pooled strains. To evaluate the reliability of this approach, we performed a pooled competition assay under one of our environmental conditions—M9, which we also used in our previous study (Acar Kirit et al. 2020). The strong correlation between the fitness effects of genes obtained from individual competition assays using flow cytometry and from pooled competition assays using HTS allowed us to use the HTS technique as a valid alternative (fig. 1, *F*_1, 42_ = 461, *r*^2^ = 0.92, *P* < 0.001). To emphasize the benefit of this approach, one HTS competition experiment with 6 replicates allowed us to compare the fitness of all 44 mutant strains in one environment simultaneously and without a loss in precision, which would otherwise have required 44 flow cytometry competition assays (each mutant vs. the wild type) done in 32 replicates each. Resulting selection coefficients of all replicates are given as part of supplementary material data S1, Supplementary Material online and plotted in Supplementary fig. 1. An additional benefit of this approach is that it more closely reflects the reality where multiple genotypes co-exist and compete against each other, as opposed to only the wild type and one mutant existing at a time.

### Environment Alters the Fitness Effects of Genes

**Fig. 2.**
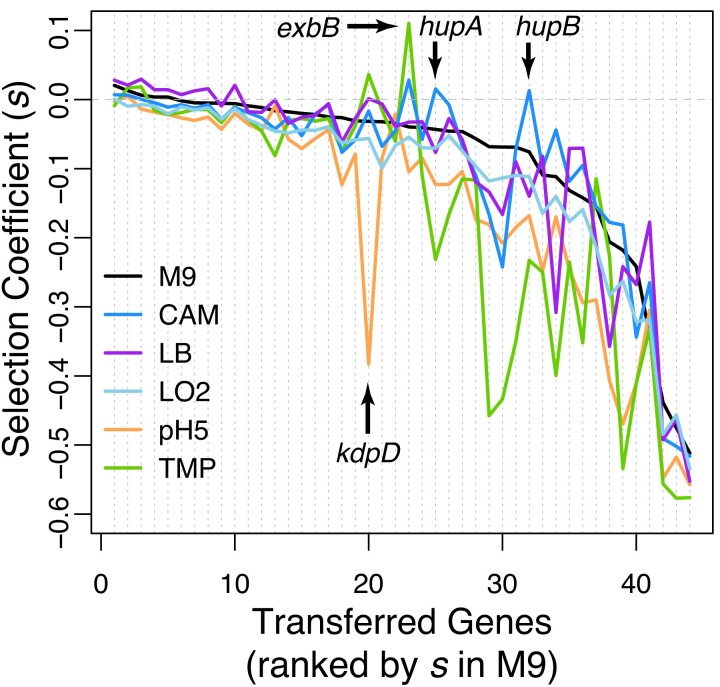
Selection coefficients of the transferred genes in six different environments, with named examples of genes whose effect changes conditionally. Transferred genes are ranked by their selection coefficient in M9 environment. Colors indicate the six environments used in this study.

We investigated if the environment affected the fitness effects of transferred genes and found a strong interaction between individual genes and the environment (fig. 2, *F*_215, 1276_ = 82, *P* < 0.001). In fact, all possible pairwise comparisons of the gene-by-environment interaction between the six environments were significant (supplementary material table S1, Supplementary Material online). The complex interactions between fitness effects and the environment resulted in a seemingly unpredictable distribution of fitness effects across environments, as the fitness rank order of genes changed drastically between environments (fig. 2). The high variability of fitness effects between environments implies that, if the environment is heterogeneous or fluctuates, even genes that are on average highly deleterious can get fixed in the population, as long as the gene is neutral or beneficial in some of the environments.

The specific function of transferred genes often explained the observed gene-by-environment interactions (fig. 2). For example, *exbB* is known to be beneficial in the presence of antibiotics, as it is a subunit of a TonB-dependent energy transduction complex that provides energy to an efflux pump (*mtrCDE*) involved in multidrug resistance (Zhao et al. 1998; Amaral 2011). Similarly, *hupA* and *hupB* are subunits of the same DNA binding protein and are known to provide fitness benefits in CAM (Kano et al. 1986, 1987). Furthermore, *kdpD* is part of a histidine kinase/response regulator system that senses K^+^ limitation and induces the *kdpFABC* operon encoding a high-affinity K^+^ uptake complex, which becomes vital under low pH conditions (Yan et al. 2011; Heermann et al. 2014). The *S.* Typhimurium ortholog seems to interfere with this function and decreases the fitness of the host cell dramatically in pH5.

The fitness effects of transferred genes can be a consequence of the transferred copy altering the effective gene dosage in the new host (Couturier and Rocha 2006; Bershtein et al. 2015). We have previously shown for this same set of genes (grown in M9 only) that deleterious effects often arose as a consequence of changes in gene dosage resulting from an introduction of a homologous gene copy (Acar Kirit et al. 2020). Here, we adopted a different approach to assessing whether dosage drove the observed fitness effects, relying on RNA-seq data. In contrast to our previous work, we did not find a significant relationship between the fitness effects and the difference in relative expression levels of newly transferred genes and their endogenous orthologs, suggesting dosage itself was not the main driver of the observed trends in fitness effects (supplementary material fig. S2 and supplementary material data S3 and S4, Supplementary Material online).

### Selective Barriers to HGT do not Determine Gene-by-Environment Interactions

While gene function can explain the gene-by-environment interactions for at least some genes used in this study, we wanted to understand if more general factors determined the fitness effects of genes in each environment. Specifically, we focused on the major selective barriers to HGT—the functional category of the transferred genes (Rivera et al. 1998; Jain et al. 1999); the number of protein–protein interactions (PPI) (Jain et al. 1999; Cohen et al. 2011); the divergence between the donor and the recipient cell that is inferred as a difference in their GC content or the codon usage bias (Drummond and Wilke 2009; Tuller et al. 2011; Baltrus 2013); gene length (Lynch and Marinov 2015). These barriers have been previously identified for horizontally transferred genes through bioinformatics approaches, and we previously conducted the first experimental test for their role in determining fitness effects systematically across many transferred genes in a single environment (M9) (Acar Kirit et al. 2020). Here, individually in each of the six environments, we conducted a multiple linear regression to test whether any of these barriers determined the observed fitness effects. We found that only gene length significantly correlated with fitness effects in environments pH5, TMP and CAM, with longer genes being associated with lower fitness (supplementary material data S6 and supplementary material table S2, Supplementary Material online).

### Fitness of Nearly Lethal and Nearly Neutral Genes Is More Consistent across Environments

The inability to identify general factors and selective barriers that drive gene-by-environment interactions implies that the fitness effect of a transferred gene is driven predominantly by its function in a given environment. Does that mean that, if we know the fitness effect of a gene in one environment, we can never assume its fitness effect in another environment and instead must measure those effects directly?

**Fig. 3.**
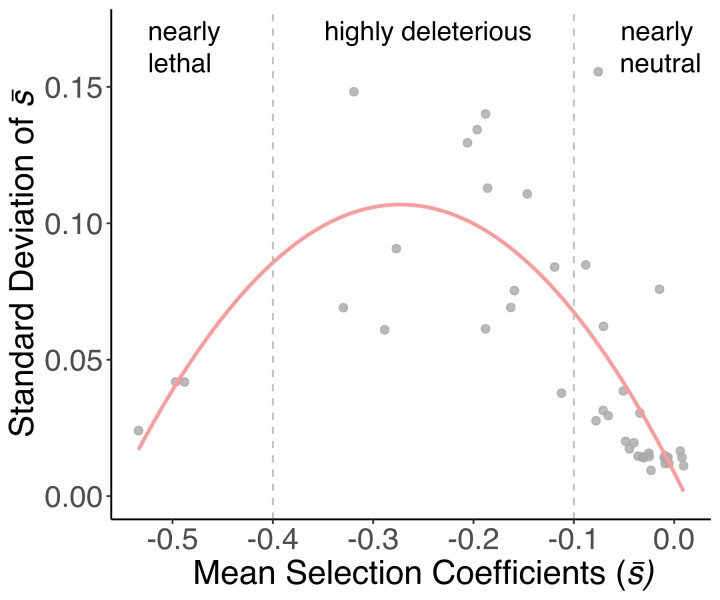
Gene-by-environment interaction is strongest for highly deleterious genes. Relationship between mean and standard deviation of selection coefficients of newly transferred genes over all environments. Solid line is the quadratic correlation (F_2, 41_ = 32.160, *r*^2^ = 0.611, *P* < 0.001).

To address the question of whether knowing the fitness effects in one environment can be used to assume fitness effects in other environments, we studied the relationship between fitness effects of each gene across all tested environments. Genes with intermediate average fitness effects over all environments (selection coefficient, −0.4 < *s̅* < −0.1) have a less consistent fitness effect that is more likely to vary between environments (fig. 2). This is true irrespective of which environment we use to rank the order of genes (supplementary material fig. S3, Supplementary Material online). Indeed, we observed a significant quadratic relationship between mean fitness effects and their standard deviation across the six environments (*F*_2, 41_ = 32.71, *r*^2^ = 0.596, *P* < 0.001) (fig. 3). This relationship was observed even when the environments were analyzed individually, suggesting a generalizable property of transferring genes into a new host (supplementary material table S3, Supplementary Material online). We divided the tested genes into three categories: 1) “nearly neutral” (*s̅* > − 0.1); 2) “highly deleterious” (− 0.1 > *s̅* > − 0.4); and 3) “nearly lethal” (*s̅* < − 0.4) (fig. 3). Only the “highly deleterious” genes exhibited strong environmental dependence, while the fitness effects of “nearly neutral” and “nearly lethal” genes were consistent and less influenced by the environment. Note that, however, this does not guarantee that every horizontally transferred gene would follow this same trend, especially if there is not an endogenous ortholog in the recipient cell or if they are silenced due to any of the numerous mechanisms that evolved to compensate the risks of acquiring genes horizontally.

The existence of these three distinct groups can at least in part be explained by the specific functions of genes in question. The low environmental variability of fitness effects of “nearly lethal” genes (excision nuclease subunit C *uvrC*, outer membrane lipoprotein carrier protein *lolA*, and DNA topoisomerase III *topB*) likely arose from the vital role these genes play in the cell. Similarly, some of the “highly deleterious” genes are beneficial in some of the tested environments because of their function, such as the energy transducing Ton system subunit *exbB*, DNA-binding transcriptional dual regulator HU proteins *hupA* and *hupB* that contribute to antibiotic resistance (Kano et al. 1986, 1987; Zhao et al. 1998; Amaral 2011). The relationship between gene-by-environment dependence and gene function of the “nearly neutral” genes is less clear.

We attempted to better understand the potential differences between these three groups of genes. Specifically, we examined if the genes in the groups differed based on the number of interaction partners, length of the coding sequence, level of divergence between orthologs, difference in the codon bias between orthologs, and the change in the expression level of the endogenous copy of the gene across all environments. The genes in the three tested groups did not differ significantly based on any of these factors (supplementary material table S4, Supplementary Material online). The “highly deleterious” group was also not enriched with informational or operational genes (8 informational out of 17 “highly deleterious” genes, compared with 10 of 27 genes for “nearly neutral” and “nearly lethal” genes, Fisher exact test, *P* = 0.544).

### The Distribution of Fitness Effects has Same Shape Across all Environments

The distribution of fitness effects across all genes (DFE) is a fundamental property in genetics that determines how a population might respond to selection (Eyre-Walker and Keightley 2007). The shape of the distribution captures the average effect of a novel mutation or a newly transferred gene and the frequency of deleterious, neutral, and beneficial mutations or transferred genes. Knowing the shape of the DFE is crucial to understanding and predicting evolution (Soskine and Tawfik 2010). While DFEs have been experimentally characterized for a wide variety of biological systems and organisms (Sanjuán et al. 2004; Eyre-Walker et al. 2006; Firnberg et al. 2014; Sarkisyan et al. 2016), only a handful of studies looked at the relationship between horizontally transferred genes and the environment, focusing on plasmids containing multitude genes (San Millan et al. 2018; Alonso-Del Valle et al. 2021). Consequently, little attention has been given to how environment affects and alters DFEs of horizontally transferred individual genes.

**Fig. 4.**
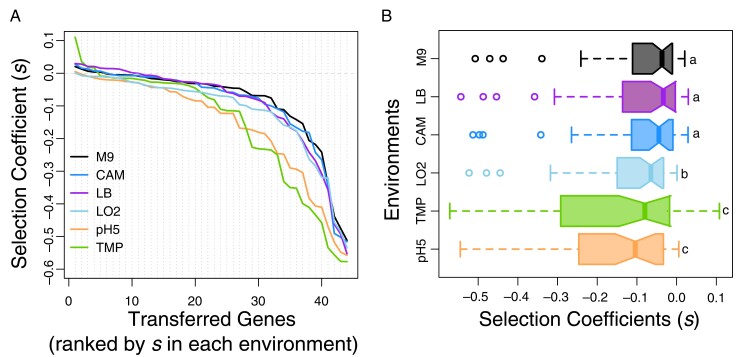
The shape and spread of DFEs is not affected by the environment. Selection coefficients of the transferred genes in six environments. (*A*) Lines connect genes measured in the same environment, and show the overall shape of the DFEs. Genes are ranked according to their selection coefficients in the environment that they were measured in. (*B*) Boxplot representation of DFEs, demonstrating that the average fitness effect of transferred genes changed between environments, even if the shape and spread of the DFEs did not. Letters a, b, and c indicate the three classes of DFEs, identified by the Wilcoxon signed ranks pairwise test.

In spite of the strong gene-by-environment interaction (fig. 2), we found that the overall shape and spread of the DFEs remained unchanged by the environment (fig. 4*A*, Kolmogorov–Smirnov test, supplementary material table S5, Supplementary Material online). The only aspect of the distributions affected by the environment was the median fitness effects across all transferred genes, meaning that DFEs were systematically shifted. Testing for the central tendency (median, Wilcoxon signed rank) of DFEs in the six environments identified three classes of DFEs (fig. 4*B*, supplementary material table S6, Supplementary Material online). This shift was not due to a common, environmentally imposed stress, as in that case medians of the DFEs would be identical because they were all measured relative to the wild type. In fact, for DFEs to exhibit the observed shifts in central tendencies (fig. 4*B*) while maintaining the same overall shape and spread, environments must amplify the cost of introduced genes in a systematic manner. One possible explanation is that under more stressful conditions cells may be less tolerant to the additional stress of an acquired gene. We did not find evidence for this hypothesis, as the central tendency of DFEs did not scale with the severity of the environmental stress (supplementary material table S7, Supplementary Material online, *F*_1,4_ = 1.887, *r*^2^ = 0.15, *P* = 0.242, supplementary material fig. S4, Supplementary Material online).

Taken together, these results suggest that although affected the fitness effects of individual genes, the overall shape and spread of DFEs were consistent across environments. The shape and spread of DFEs of horizontally transferred genes might therefore be a universal property, with changes in the environment altering only the median fitness effect of transferred genes.

## Discussion

Here, we employed HTS to assess the competitive fitness of horizontally transferred and expressed orthologous genes under different physiologically relevant stress environments. Previous uses of HTS for fitness assessment revealed the potential of the technique; however, being able to sequence only the horizontally transferred genes from an otherwise isogenic population provided us with extra advantages. For instance, we did not need to modify our host organisms with transposons or barcodes prior to pooled competitions (Levy et al. 2015; Venkataram et al. 2016; Kinsler et al. 2020; Fasanello et al. 2020; Couce et al. 2022), nor did we have to sequence their whole genomes (Barrick et al. 2009; Lang et al. 2013).

Environment is a major factor in evolution, shaping the fitness effects of mutations (Hietpas et al. 2013; Flynn et al. 2020), viruses (Lalić et al. 2011), and newly acquired plasmids (San Millan et al. 2018). Here, we examined the role of environment on the fitness effects of horizontally transferred orthologous genes and found that the fitness effects of horizontally transferred genes are highly dependent on the environment, with abundant gene-by-environment interactions indicating that, in the long run, success of an HGT event is determined by the heterogeneity of the environment. How environmental change alters the fitness effect of a gene can be at least partially inferred if the fitness effect in one environment is known, as it does not depend only on the gene's specific function but also on its overall fitness effect (fig. 3). Namely, nearly lethal genes are likely to remain nearly lethal across all environments, as are the neutral genes. It is the genes with an intermediate overall fitness effect that are most affected by the environment, exemplifying large variations in fitness across the tested conditions. Genes with a stronger gene-by-environment interaction may segregate in the population longer, depending on how heterogeneous the environment is or how rapidly it fluctuates. In other words, the likelihood of a successful HGT event does not necessarily correlate with the average fitness effect of a gene.

Certain intrinsic properties of transferred genes also impact their fitness effects independently of environmental change, forming the selective barriers to HGT. Previously, we showed that the fitness effects of this same set of transferred orthologs in a single environment (M9) were in part determined by their length (Acar Kirit et al. 2020). Here, we showed that longer genes were associated with lower fitness in some, but not all tested environments. We have also previously identified gene dosage as another factor driving the fitness effects of transferred genes (Acar Kirit et al. 2020). Here, using a different approach based on RNA-seq, we observed that the relative increase in expression levels due to the introduction of a homologous copy of a gene did not correlate with the observed fitness effects in any environment (supplementary material fig. S2, Supplementary Material online). While this observation suggests dosage was not the primary factor driving the observed fitness effects, further investigations are needed to properly identify its role in horizontal transfer of orthologs. Methodological differences between our previous and this study likely resulted in the different estimates of the importance of dosage on the fitness effects of transferred genes. We have previously (Acar Kirit et al. 2020) studied dosage as a fitness effect associated with having a second copy of the same gene, rather than as a consequence of differences in expression levels of orthologous genes. This discrepancy points to the need for developing more consistent techniques for measuring dosage effects. Other barriers to HGT, such as codon usage and GC content, are unlikely to drive the differences in fitness effects between genes and especially their environment dependence, as orthologs from closely related species typically exhibit only modest differences in GC content and codon usage (Acar Kirit et al. 2020). Finally, the observed patterns of fitness effects in HGT between closely related organisms might also arise due to genetic dominance between orthologous genes (Rodríguez-Beltrán et al. 2020).

More broadly, trying to explain HGT only by considering the barriers to transfer, as is often done (Jain et al. 1999; Wellner et al. 2007; Cohen et al. 2011; Tuller et al. 2011; Bershtein et al. 2015), runs a risk of severely underestimating the critical role of the environment in determining the outcomes of HGT. Similarly, our study points to the potential limitations of experimentally observing HGT in only a single environment, as has predominantly been done so far (Knöppel et al. 2014; Bershtein et al. 2015). Understanding the evolutionary likelihood of a successful horizontal transfer event must be viewed across a range of biologically meaningful environments while still considering the mechanistic nature of the gene's role in the recipient cell.

In this study, we examined how environment shaped the fitness effects of genes transferred to a closely related organism where an orthologous copy is already present in their new host. This is a specific scenario that does not capture all the complexity of HGT. While this scenario might be common (Adato et al. 2015), an orthologous copy might not always be present in the host. The fitness effects of such new genes would depend primarily on whether they introduce a new function to the host, as opposed to the dosage effect for example, and hence the factors that shape their evolutionary fate might be quite different. Similarly, we adopted a very simplified genetic scenario, where all transferred genes were in the same genetic context and expressed through the same promoter. The reality, of course, can be much more varied, with transferred genes finding themselves flanked by mobile elements, or integrated into the chromosome as opposed to being on a plasmid, etc. We adopted a controlled experimental approach to study one such scenario in detail. Extending our findings to different sets of genes under different genetic conditions will be key to developing a deeper understanding of the factors that shape fitness effects and, hence, HGT.

Despite the fitness effects of genes being highly environment-dependent, overall DFEs are consistent in shape and spread across all environments. This finding enables and justifies more robust modeling of HGT, as modeling and predicting evolution requires assuming an underlying DFE (Eyre-Walker and Keightley 2007). In other words, while predicting the evolutionary fate of a specific transferred gene is obscured by myriad factors that determine its fitness effect across environments, the overall, average fate of populations might be more predictable and robust.

## Materials and Methods

### Strains and Plasmids

We used *E. coli* K12 MG1655 (DSM18039) strain with the chromosomal insertion of a *tetR* cassette (Acar Kirit et al. 2020). This cassette contained the gene for the repressor protein tetR that regulates the expression of the transferred genes under control of the constitutive promoter P_N25_, and spectinomycin resistance. The cassette was inserted at the λ-att site of *E. coli* chromosome by using a modified version of pZS4Int1 plasmid and the helper plasmid pLDR8 carrying lambda integrase, as described before (Lutz and Bujard 1997).

All 44 *S.* Typhimurium genes were cloned under the inducible P_LtetO-1_promoter into the low copy number (3–4 plasmids/cell) pZS* plasmid backbone of Lutz & Bujard, which carried Ampicillin resistance cassette and hence all the environments were supplemented with Ampicillin 50 µg/mL (Lutz and Bujard 1997; Acar Kirit et al. 2020).

### Culture Conditions and Environments

We chose environments that are representative of the conditions potentially experienced by *S.* Typhimurium and *E. coli* species. Rich M9 medium contains 1 × M9 salts (Sigma-Aldrich, Cat no. M6030), 1% CAA (Sigma-Aldrich, Cat no. A2427), 0.4% glucose, 2 mM MgSO_4_, 0.1 mM CaCl_2_. To estimate gene expression, we inserted *gfp* under P_LtetO-1_promoter. Gene expression was induced using anhydrotetracycline (aTc), and using flow cytometry, we have adjusted the aTc concentration to reach similar levels of GFP fluorescence in each environment. All 250 mL cultures for sequencing were grown in 500 mL flasks at 37° C and 180 rpm (except for the LO_2_ condition) in a water bath and supplemented with ampicillin 50 µg/mL as the pZS* plasmid backbone had Ampicillin resistance gene. We used rich M9 supplemented with ampicillin 50 µg/mL, at pH 7 with aTc 5 ng/mL as the standard medium “M9.” The wild type has a reciprocal time (*μ*) of 0.0173 min^−1^ in this standard medium. Other tested growth conditions are described as: “CAM”—M9 rich medium supplemented with ampicillin 50 µg/mL and CAM 1.2 µg/mL and aTc 5 ng/mL, *μ*_CAM_ = 0.0087 min^−1^; “LB”—Lennox broth and aTc 12 ng/mL, μ_LB_ = 0.0289 min^−1^; “LO_2_”—M9 rich medium supplemented with ampicillin 50 µg/mL and overlaid with paraffin oil to create a LO_2_ condition and aTc 5 ng/mL, μ_LO2_ = 0.0116 min^−1^; “pH5”—M9 rich medium at pH5 supplemented with ampicillin 50 µg/mL and aTc 5 ng/mL, *μ*_pH5_ = 0.0116 min^−1^; “TMP”− M9 rich medium supplemented with ampicillin 50 µg/mL, TMP 0.3 µg/mL and aTc 4 ng/mL, *μ*_TMP_ = 0.0087 min^−1^ (supplementary material table S7, Supplementary Material online).

### Selected Genes

The set of genes, plasmid constructs, and bacterial strains used in this study are derived from a previous study (Acar Kirit et al. 2020). Briefly, in total 44 genes from *Salmonella enterica* subsp. *enterica* serovar Typhimurium LT2 genome (DSM18522, Genbank AE006468.1 [McClelland et al. 2001]) were chosen arbitrarily avoiding genes that are known to be related to mobile genetic elements, such as phage proteins, transposable elements, or insertion sequences, as well as ribosomal and transfer RNAs. During selection several precautions were taken to avoid introducing biases in functional categories and number of interacting partners, as previously described in Acar Kirit et al. 2020. All 44 *S.* Typhimurium genes were cloned in the low copy number plasmid pZS* under P_LtetO-1_promoter. In this study, an additional random fragment of the *tetA* gene (721 bp, the mean length of all inserted genes) was cloned into the pZS* plasmid without a promoter to be used as the “wild type” in competition assays. We checked whether the addition of this fragment altered fitness by conducting a competition assay using BD FACSCanto II flow cytometer as described previously (Acar Kirit et al. 2020) (*s* = 0.001, Wilcoxon signed rank test, *P* = 0.24).

### Competition Assays and Sequencing

An additional genotype, carrying a phenotypically neutral and unique sequence in the pZS* plasmid but otherwise identical to the host cell (*E. coli* MG1655 att-λ::[tetR-Sp^R^]+att-p21::[CFP/YFP-Kn^R^]), was used as the “wild type” during these competition assays. In total 6 replicate competitions were performed across 3 different days for each environment. 1:1000 dilutions of separate overnight cultures of 45 *E. coli* clones each carrying a different plasmid were grown in 20 mL M9 rich medium to OD_600_ 0.2 and then mixed at equal volumes. A concentrated stock culture was prepared in 1 × M9 salts with 10% DMSO-Dimethyl sulfoxide. Cell concentration was verified by colony forming uint (CFU) counting of the dilutions of frozen stock. Aliquots of this “mixed stock” of 45 different clones was stored at −80°C to be used only once as inoculum for each competition. Two larger samples of this mixed stock are used to estimate the starting frequencies.

For the competition assays, 250 mL of corresponding medium was inoculated with 10^7^ cells from mixed stock for each environment tested and grown until OD_600_ 0.4. Plasmid DNA was extracted from 200 mL of this culture using ZR Plasmid Miniprep™ Kit—Classic (Zymo research, D4015).

DNA was sheared with S220 AFA™ Focused-ultrasonicator (Covaris®) to obtain a fragmentation size of 300–700 bp. The DNA library of 6 different environments (6 biological replicates each) and the initial stock (2 technical replicates) were prepared and sequenced on Illumina HiSeq 2500 (100 bp SE) by the sequencing company Vienna Biocenter Core Facilities (VBCF) NGS Unit (www.vbcf.ac.at, Vienna, Austria). In addition, quality checking and de-multiplexing of the raw data was also provided by the VBCF.

### Sequencing Data Processing and Calculation of Selection Coefficients

Sequence reads with an average read quality of ≥ 34 were retained for further analysis. Sequencing reads were mapped with GMAP (version2016.09.23–0) against a personalized reference containing FASTA files of 44 *S.* Typhimurium genes, *tetA* fragment and backbone of the pZS* plasmid, built with gmap_build function. Parameters were set to −no-chimeras −nosplicing −nofails −npaths = 0 to increase mapping accuracy (Wu and Watanabe 2005). Mapped SAM files were converted to BAM format, sorted and indexed with samtools (version 1.12) (Li et al. 2009).

In order to obtain depth per gene, bedtools (version: 2.30.0) was used with the following parameters: bedtools genomecov -d −ibam (Quinlan and Hall 2010). As the plasmid carried an ampicillin resistance gene, we used it as a control and normalization factor between replicates, as each gene copy had to always be paired with an ampicillin resistance gene copy. The read depths of genes as well as the ampicillin control gene observed in a single M9 library were substantially different from all other replicates in that environment, resulting in its exclusion from subsequent analyses (shown in red in supplementary material fig. S1-M9, Supplementary Material online) The sequencing analysis pipeline is provided as part of the Supplementary material to this paper. The raw data for this sequencing experiment is deposited in NCBI BioProject repository with accession number PRJNA867418.

Fitness effects of selected genes (*s*) were estimated for each replicate by using the regression model ln(1 + *s*) = (ln *R_t—_*ln *R_0_*)/*t*, where R is the ratio of the frequencies of mutant (depth of gene) to wild type (depth of *tetA* fragment) and *t* is the number of generations (Elena et al. 1998). Initial frequencies were obtained from the mean depth of the two replicates of the mixed stock, which is used for inoculation of each competition. Time *t* is end of the competition assay, corresponding to 8.25 generations. According to this formula, fitness effects of genes are calculated relative to that of wild type. We have estimated the number of generations by performing colony counts of serial dilution plates of the frozen aliquots of the “mixed stock” of 45 different clones that we used as inoculum for each competition experiment and the final culture that we used to isolate the plasmids at OD_600_ = 0.4 for each environment.

Calculated selection coefficients of the transferred genes for each replicate of our six different environments are given in supplementary material data S1, Supplementary Material online (excel sheet) and shown in supplementary material fig. S1, Supplementary Material online. The mean, minimum and maximum depths of each gene in each replicate are given in supplementary material data S2, Supplementary Material online (excel sheet). The mean selection coefficients and their overall mean used in fig. 3 are given in supplementary material table S8, Supplementary Material online.

### RNA-seq

We performed RNA-seq to determine the expression level of the inserted genes and of their endogenous orthologs that are already present in the host within each environment, as well as to confirm whether the potential interaction partners of each gene were expressed in each environment. The host strain contained *cfp* gene in its genome (used for fitness measurements with flow cytometry) and the sequences of *cfp* and *gfp* genes are very similar making it difficult to distinguish in sequence data. Therefore, we replaced *gfp* with *mCherry* for the RNA-seq experiment. Cells were grown under the same conditions as competition assays, where the starter stock (inoculation of 10^7^ cells) only included *mCherry* containing strain instead of the mixed stock of 45 strains. Growth was stopped by adding Qiagen RNA protect Bacteria Reagent (cat no. 76506) onto 20 mL of cultures at OD_600_ 0.4. Total RNA preparation after this point was performed as described previously (Acar Kirit et al. 2020). Library preparation (RiboZero, NEB), further quality checks and HTS (HiSeq2500-v4, SR100 mode) were performed at the VBCF NGS Unit (www.vbcf.ac.at). The data is deposited in NCBI BioProject repository with accession number PRJNA866657, and abundance data is given as supplementary material data S3, Supplementary Material online.

Briefly, RNA-Seq data quality control was done using the Adapter Removal program (ver. 2.3.1, Schubert et al. 2016) with the parameters “–trimns –maxns 1 –trimqualities –minquality 30.” RNA-Seq alignment and quantification were done using the Salmon (v1.6.0, Patro et al. 2017) with parameters “salmon quant -l A –validateMappings,” using the reference genome of *E. coli* strain MG1655 from NCBI (genome sequence GenBank ID U00096.3).

RNA-seq data also served to inspect whether all of the listed interaction partners (PPI) of the 44 selected genes were expressed under our experimental conditions. Interacting gene partners for each gene were identified as previously described (Acar Kirit et al. 2020). Briefly, using the interaction partner information provided in table S8 in Hu et al (2009) we have identified the potential partners of each of our 44 transferred genes with > 0.70 integrated score in *E. coli*. The table with each gene and their interacting partners with the abundance values in each environment is given in supplementary material data S5, Supplementary Material online. After obtaining the expression levels for the whole transcriptome from RNA-seq data (supplementary material data S3, Supplementary Material online), to determine whether a gene was expressed or not, we needed a threshold level of expression below which a gene would be eliminated from further analyses. To this end, we inspected the expression levels of genes that are known as being repressed under our experimental conditions, that is lactose operon and arabinose regulon genes. Expression level of these genes ranged from 0.5–10 TPM in our RNA-seq data. Based on this observation and previous similar studies, we set a threshold value of TPM ≥ 10 when counting a PPI partner as expressed (Acar Kirit et al. 2020). This information was then used as one of several intrinsic properties of the transferred genes to investigate their potential effect on fitness. Results of these analyses are given in supplementary material table S2, Supplementary Material online, and the data used for the multiple regression analysis is given in supplementary material data S6, Supplementary Material online, with the details of the analysis are given under the statistical analysis section below.

### Statistical Analyses

In order to estimate differences between environments a paired and two-sided Wilcoxon signed-rank test was performed with *α* = 0.05 on selection coefficients of genes in each pairwise comparison of six environments. In order to test if the shapes of distributions of environments were different, two-sample and two-sided Kolmogorov–Smirnov tests were performed (Lehmann and D'abrera 2006). In both of these tests, selection coefficients of the genes in each environment were represented by the mean of 5 or 6 biological replicates, and final *P*-values were corrected for multiple testing using BH-FDR method (Benjamini and Hochberg 1995).

To investigate the interaction between environment and genes, a repeated measures analysis of variance (ANOVA) test was performed by using selection coefficients of the genes composed of 5 or 6 replicates for each environment, with the formula: Selection Coefficients ∼ Environment * Gene + within replicates (replicates). We applied this test on each pairwise comparison of six environments, and final *P*-values were corrected for multiple testing using BH—FDR method (Benjamini and Hochberg 1995).

Furthermore, the mean and standard deviation of the selection coefficients of each gene across all environments were calculated. We investigated the relationship between the mean and standard deviation of the selection coefficients of the transferred genes by performing linear regressions with the formulas: standard deviation ∼ mean and standard deviation ∼ mean + mean^2^. We observed a significant linear relationship between the mean and standard deviation of fitness effects of genes across the six environments (F_1, 42_ = 8.181, *P* = 0.007, *r*^2^ = 0.143). A quadratic model, however, explains the data better (F_2, 41_ = 32.71, *P* < 0.001, *r*^2^ = 0.596), suggesting a bell-shaped relationship between mean fitness effects of the genes and variance of them across all environments (fig. 3).

To investigate the role of intrinsic factors on fitness effects of transferred genes, we employed multiple linear regression. After investigating interactions between variables through a maximal model, we used model simplification to arrive at the following model utilized separately for each environment: “*S.* Typhimurium selection coefficients” ∼ “Functional Category (as dummy variable)” + “PPI level” + “Deviation in GC% between orthologs” + “Deviation in codon usage between orthologs” + “Gene length in nucleotides.” Furthermore, genes were split into three groups: 1) “nearly neutral” (s > − 0.1); 2) “highly deleterious” (− 0.1 > s > − 0.4); and 3) “nearly lethal” (s < − 0.4), based on the mean fitness effects of genes between environments. We inspected if these three groups could be separated from each other by the means of several intrinsic properties of the transferred genes (supplementary material table S4, Supplementary Material online, number of interaction partners (PPI), length of the coding sequence, difference in the GC content between homologs, difference in the codon bias between homologs (FOP), and the change in the level of the expression of the endogenous copy of the gene over all conditions (TPM). The data used for these analyses is given in supplementary material data S6, Supplementary Material online.

We performed separate one-way ANOVA tests for each of these properties with the formula: Gene property (dependent continuous variable) ∼ genegroup (independent factorial variable, 3 levels). A statistically significant difference was considered at *P* < 0.05. An additional Fisher's exact test was performed to examine whether “highly deleterious genes” were enriched for the functional category of the genes as Informational and Operational genes compared with the rest of the genes.

All statistical analyses were performed in the R software package (version 4.1.2) and RStudio (version 2022.02.1).

## Supplementary Material

msac220_Supplementary_Data

## Data Availability

Authors confirm that all relevant data are included in the article and/or its supplementary information files. Furthermore, the raw data for the plasmid sequencing experiment is deposited in NCBI BioProject repository with accession number PRJNA867418, and the raw RNA-seq data is deposited in NCBI BioProject repository with accession number PRJNA866657.
