# Supplementary material for: The Role of the Environment in Horizontal Gene Transfer
Source: Mol Biol Evol. 2022 Oct 13;39(11):msac220. doi: 10.1093/molbev/msac220 (PMC9641970; doi:10.1093/molbev/msac220)
Supplement: msac220_Supplementary_Data [file msac220_supplementary_data.zip › SupplementaryMaterial_EnvHGT/Suppl_Tables_Figures.pdf]

Supplementary Material

Supplementary Table 1. *Analysis of Variance of the pairwise comparisons of environments.*

**Pairwise comparisons**		**Environment**	**Gene**	**Gene X Environment**
**M9**	**CAM**	$F_{1,396} = 72$<br>p<.001	$F_{43,396} = 2534$<br>p<.001	$F_{43,396} = 80$<br>p<.001
**M9**	**LB**	$F_{1,396} = 90$<br>p<.001	$F_{43,396} = 1987$<br>p<.001	$F_{43,396} = 83$<br>p<.001
**M9**	**LO2**	$F_{1,396} = 292$<br>p<.001	$F_{43,396} = 562$<br>p<.001	$F_{43,396} = 3.5$<br>p<.001
**M9**	**pH5**	$F_{1,396} = 4837$<br>p<.001	$F_{43,396} = 1687$<br>p<.001	$F_{43,396} = 108$<br>p<.001
**M9**	**TMP**	$F_{1,396} = 2731$<br>p<.001	$F_{43,396} = 1075$<br>p<.001	$F_{43,396} = 143$<br>p<.001
**CAM**	**LB**	$F_{1,440} = 5.27$<br>p=.022	$F_{43,440} = 2230$<br>p<.001	$F_{43,440} = 127$<br>p<.001
**CAM**	**LO2**	$F_{1,440} = 202$<br>p<.001	$F_{43,440} = 681$<br>p<.001	$F_{43,440} = 19.2$<br>p<.001
**CAM**	**pH5**	$F_{1,440} = 4510$<br>p<.001	$F_{43,440} = 1896$<br>p<.001	$F_{43,440} = 166$<br>p<.001
**CAM**	**TMP**	$F_{1,396} = 2650$<br>p<.001	$F_{43,396} = 1257$<br>p<.001	$F_{43,396} = 162$<br>p<.001
**LB**	**LO2**	$F_{1,440} = 155$<br>p<.001	$F_{43,440} = 669$<br>p<.001	$F_{43,440} = 20.8$<br>p<.001
**LB**	**pH5**	$F_{1,440} = 3595$<br>p<.001	$F_{43,440} = 1712$<br>p<.001	$F_{43,440} = 119$<br>p<.001
**LB**	**TMP**	$F_{1,440} = 2268$<br>p<.001	$F_{43,440} = 1213$<br>p<.001	$F_{43,440} = 126$<br>p<.001
**LO2**	**pH5**	$F_{1,440} = 733$<br>p<.001	$F_{43,440} = 683$<br>p<.001	$F_{43,440} = 34$<br>p<.001
**LO2**	**TMP**	$F_{1,440} = 614$<br>p<.001	$F_{43,440} = 615$<br>p<.001	$F_{43,440} = 77$<br>p<.001
**pH5**	**TMP**	$F_{1,440} = 1.25$<br>p=.264	$F_{43,440} = 1175$<br>p<.001	$F_{43,440} = 133$<br>p<.001
**OVERALL**		$F_{5,1276} = 1251$<br>p<.001	$F_{43,1276} = 2971$<br>p<.001	$F_{215, 1276} = 82$<br>p<.001

F statistics and p-values from pairwise comparisons of all environments with a repeated measures ANOVA. Shaded fields are significant at α = 0.05, values are corrected for multiple testing by FDR. Each of the 44 genes are represented by 5 or 6 replicates per environment.

Supplementary Table 2. *Analyses of the selective barriers on HGT in six different environments.*

	**Functional Category**	**Prot-Prot Interactions**	**Deviation in GC**	**Deviation in FOP**	**Gene Length**
**M9**	0.3858	0.3074	0.383825	0.2075455	0.169125
**PH5**	0.4966756	0.169125	0.2727064	0.107976	**0.01011**
**TMP**	0.69884	0.675031	0.3174375	0.383825	**0.0436**
**CAM**	0.33695	0.2424692	0.621	0.383825	**0.0436**
**LB**	0.383825	0.3563684	0.4725	0.2075455	0.169125
**LO2**	0.33695	0.2424692	0.383825	0.2075455	0.107976

Column represent factors given as explanatory variables in a multiple regression model, and p- values are listed for that factor in each environment. Significant values are shown in bold at α = 0.05, values are corrected for multiple testing by FDR. GC: GC content of the DNA sequence; FOP: difference in the codon bias between homologs.

Supplementary Table 3. *Quadratic model fitted to the fitness effects of genes in each environment and standard deviation of these effects across all environments.*

	**$F_{2,41}$**	**p-value**
**M9**	18.92	<0.001
**LB**	17.64	<0.001
**PH5**	32.6	<0.001
**TMP**	10.27	<0.001
**CAM**	6.148	0.004
**LO2**	19.35	<0.001
**Overall**	32.71	<0.001

The significant p-values indicates that genes with intermediate average cost have fitness effects that vary more between environments.

Supplementary Table 4. *Analysis of Variance for the intrinsic properties of transferred genes.*

Factors	F ratio	p-Values
GC content	0.016	0.984
PPI *	1.877	0.498
FOP	0.162	0.984
Length	4.731	0.084
TPM	1.415	0.51

See Materials and Methods for the detailed description of the test. We compared the three groups: 'nearly neutral', 'highly deleterious', and 'nearly lethal' genes for each of the factors on the table. α = 0.05, values are corrected for multiple testing by FDR. PPI: protein-protein interactions, number of interaction partners; FOP: difference in the codon bias between homologs, TPM: transcript per million from RNA-seq analysis.

** Since difference in the PPI level among environments was insignificant, mean number of PPI over all environments is used for the analysis.*

Supplementary Table 5. *Kolmogorov - Smirnov tests of the pairwise comparisons of environments testing for the difference in shape and spread.*

	**M9**	**CAM**	**LB**	**LO2**	**pH5**	**TMP**
**M9**	-	0.994	0.737	0.228	0.228	0.274
**CAM**	0.091	-	0.872	0.274	0.228	0.388
**LB**	0.159	0.136	-	0.228	0.228	0.440
**LO2**	0.273	0.250	0.273	-	0.440	0.440
**pH5**	0.318	0.273	0.273	0.205	-	0.737
**TMP**	0.250	0.227	0.205	0.205	0.159	-

p-values (upper diagonal) and D statistics (lower diagonal) from pairwise comparisons of all environments with two-sided Kolmogorov - Smirnov tests. α = 0.05, values are corrected for multiple testing by FDR.

Supplementary Table 6. *Wilcoxon signed rank tests of the pairwise comparisons of environments testing for the difference in central tendency - median.*

	**M9**	**CAM**	**LB**	**LO2**	**pH5**	**TMP**
**M9**	-	.265	.608	**<.001**	**<.001**	**<.001**
**CAM**	1.213	-	.15	**<.001**	**<.001**	**<.001**
**LB**	0.583	1.564	-	**<.001**	**<.001**	**<.001**
**LO2**	5.485	3.458	3.548	-	**<.001**	**.025**
**pH5**	5.543	5.065	5.287	4.750	-	.930
**TMP**	4.440	-3.735	4.353	-2.357	-0.128	-

p-values (upper diagonal) and Z statistics (lower diagonal) from pairwise comparisons of all environments with two-sided Wilcoxon signed rank tests. Shaded fields are significant with α = 0.05, values are corrected for multiple testing by FDR.

Supplementary Table 7. *Environments and growth rate of the 'wild type' in these environments.*

Treatment	Growth Media	Growth Rate [min^{-1}]	DFE Medians	DFE Variance
**LB**	Lennox broth	0.0289	-0.034	0.021
**M9**	M9 rich medium pH7	0.0173	-0.037	0.017
**LO2**	M9 rich medium overlaid with paraffin oil	0.0116	-0.065	0.017
**pH5**	M9 rich medium pH5	0.0116	-0.104	0.026
**CAM**	M9 rich medium 1.2 μg/mL Chloramphenicol	0.0087	-0.045	0.019
**TMP**	M9 rich medium 0.3 μg/mL Trimethoprim	0.0087	-0.078	0.036

The Growth rate (μ in reciprocal time units) of the 'wild type' in an environment is a proxy for the severity of stress under that environment. These results show the relationship between these environments as such: $\mu_{LB}>\mu_{M9}>\mu_{LO2}=\mu_{pH5}>\mu_{CAM}=\mu_{TMP}$

Supplementary Table 8. *Data used in the analyses, selection coefficients of the transferred Salmonella genes in six different environments.*

STM Gene ID	Gene Name	M9	CAM	LB	LO2	pH5	TMP	Mean	SD
STM0160	*yacL*	-0.006	-0.011	0.021	-0.013	-0.020	-0.010	-0.007	0.014
STM0172	*yadG*	-0.112	-0.044	-0.308	-0.140	-0.170	-0.399	-0.196	0.133
STM0226	*lpxD*	0.013	0.006	0.021	-0.010	0.005	0.017	0.009	0.011
STM0264	*dnaQ*	-0.046	-0.067	-0.058	-0.075	-0.104	-0.115	-0.077	0.027
STM0425	*thiI*	-0.154	-0.155	-0.207	-0.214	-0.290	-0.115	-0.189	0.062
STM0451	*hupB*	-0.076	0.013	-0.140	-0.112	-0.168	-0.233	-0.119	0.084
STM0614	*uspG*	-0.020	-0.052	-0.026	-0.044	-0.071	-0.027	-0.040	0.019
STM0629	*cspE*	-0.046	-0.007	-0.028	-0.052	-0.123	-0.166	-0.070	0.061
STM0648	*leuS*	-0.242	-0.344	-0.268	-0.321	-0.410	-0.411	-0.333	0.071
STM0703	*kdpD*	-0.032	-0.017	0.001	-0.056	-0.383	0.036	-0.075	0.154
STM0801	*ybhK*	0.006	0.000	0.029	-0.007	-0.014	0.018	0.006	0.016
STM0806	*moaE*	0.004	-0.011	0.014	-0.021	-0.022	-0.021	-0.010	0.015
STM0831	*dps*	-0.041	-0.058	-0.032	-0.070	-0.085	-0.110	-0.066	0.029
STM0943	*cspD*	-0.034	-0.045	-0.039	-0.067	-0.021	-0.060	-0.044	0.017
STM0945	*clpA*	-0.026	-0.076	-0.062	-0.063	-0.123	-0.075	-0.071	0.031
STM0961	*lolA*	-0.475	-0.502	-0.462	-0.456	-0.517	-0.577	-0.498	0.045
STM1061	*rlmL*	0.020	0.007	0.028	0.001	-0.003	-0.009	0.007	0.014
STM1112	*cbpA*	-0.005	-0.028	-0.009	-0.028	-0.043	-0.033	-0.025	0.014
STM1185	*rne*	-0.069	-0.242	-0.166	-0.114	-0.208	-0.434	-0.205	0.128
STM1196	*acpP*	-0.142	-0.095	-0.070	-0.159	-0.294	-0.352	-0.185	0.113
STM1298	*topB*	-0.438	-0.491	-0.492	-0.485	-0.548	-0.556	-0.502	0.044
STM1334	*infC*	-0.002	-0.007	0.007	-0.010	-0.026	-0.019	-0.010	0.012
STM1366	*ydiI*	-0.005	-0.013	0.013	-0.015	-0.030	-0.014	-0.011	0.014
STM1696	*sapF*	-0.005	-0.007	0.016	-0.012	-0.025	-0.015	-0.008	0.014
STM1946	*uvrC*	-0.512	-0.516	-0.552	-0.534	-0.557	-0.576	-0.541	0.025
STM2388	*fadJ*	-0.218	-0.182	-0.242	-0.263	-0.470	-0.534	-0.318	0.146
STM2543	*iscS*	-0.032	-0.068	-0.006	-0.099	-0.085	-0.016	-0.051	0.038
STM2643	*srmB*	-0.341	-0.265	-0.177	-0.319	-0.305	-0.332	-0.290	0.061
STM3143	*hybG*	0.004	-0.004	0.014	-0.010	-0.018	-0.016	-0.005	0.012
STM3159	*exbB*	-0.040	0.028	-0.033	-0.054	-0.104	0.110	-0.015	0.075
STM3215	*yqjI*	-0.056	-0.109	-0.119	-0.096	-0.173	-0.117	-0.112	0.038
STM3282	*pnp*	-0.131	-0.118	-0.071	-0.177	-0.246	-0.235	-0.163	0.069
STM3514	*malP*	-0.068	-0.166	-0.134	-0.118	-0.181	-0.458	-0.187	0.138
STM3656	*glyQ*	-0.016	-0.043	0.000	-0.046	-0.009	-0.081	-0.032	0.030
STM3682	*selB*	-0.109	-0.100	-0.082	-0.165	-0.248	-0.250	-0.159	0.075
STM3689	*yibL*	-0.031	-0.058	-0.027	-0.058	-0.079	-0.038	-0.049	0.020
STM3808	*ibpB*	-0.012	-0.026	-0.018	-0.037	-0.045	-0.046	-0.031	0.014
STM3854	*pstB*	-0.069	-0.072	-0.090	-0.110	-0.185	-0.350	-0.146	0.109
STM4170	*hupA*	-0.043	0.015	-0.076	-0.071	-0.123	-0.231	-0.088	0.084
STM4237	*lexA*	-0.206	-0.178	-0.357	-0.283	-0.407	-0.227	-0.276	0.090
STM4361	*hfq*	-0.025	-0.007	-0.006	-0.039	-0.044	-0.027	-0.025	0.016
STM4394	*rplI*	-0.022	-0.021	-0.023	-0.045	-0.056	-0.031	-0.033	0.015
STM4458	*ridA*	-0.009	-0.019	-0.018	-0.029	-0.036	-0.029	-0.023	0.010
STM4558	*rimI*	-0.018	-0.026	-0.035	-0.048	-0.057	-0.030	-0.036	0.015

Mean is the mean selection coefficient of the transferred genes across all environments, and SD is the standard deviation of selection coefficient of the transferred genes across all environments.

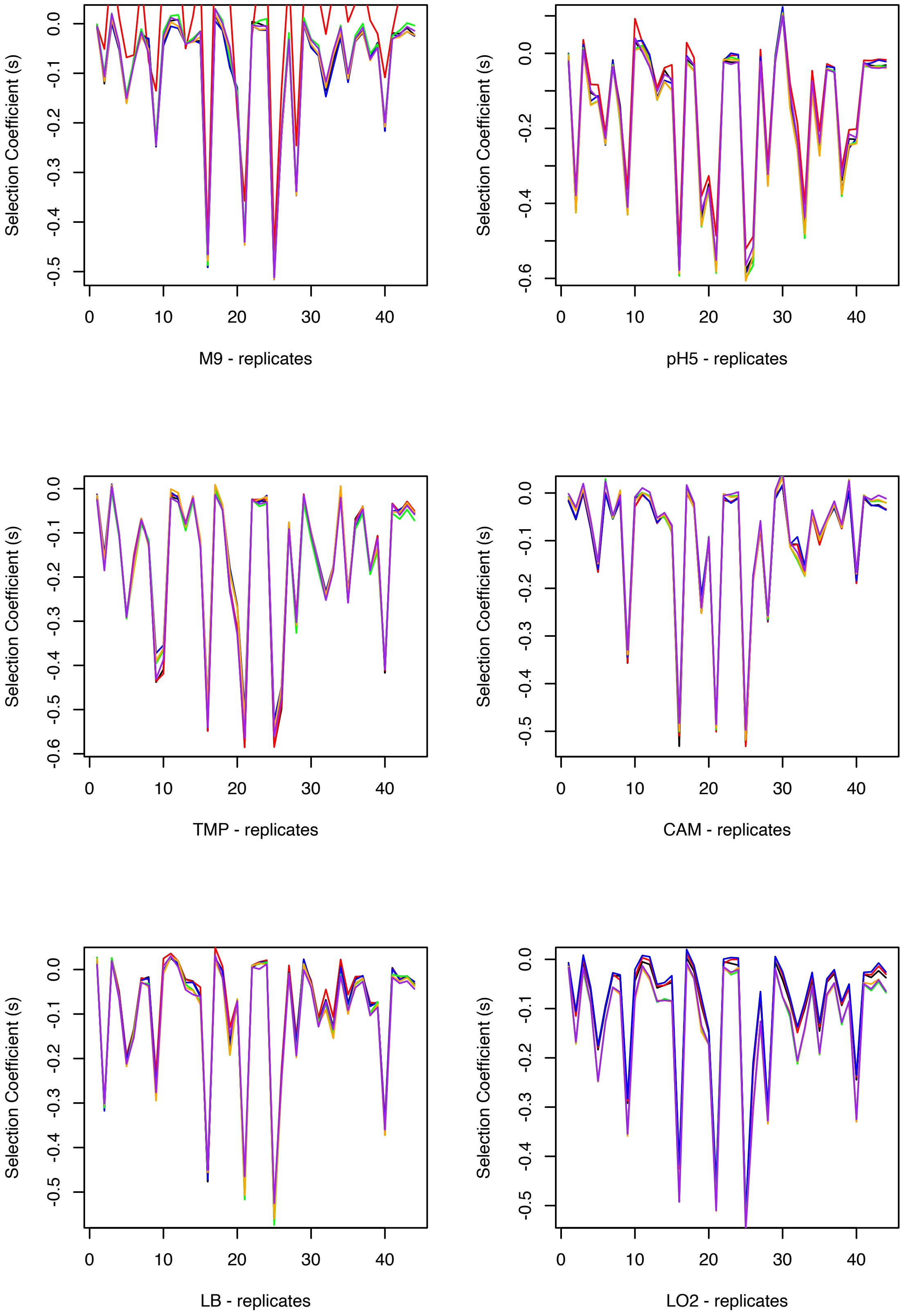

Supplementary Figure 1. *Selection coefficients of the replicates for each environment. Transferred genes are ranked by their unique STM identifiers. Colors indicate the six replicates used in this study. Note that in M9 environment, one replicate gave inconsistent results (red) and was removed from subsequent analyses.*

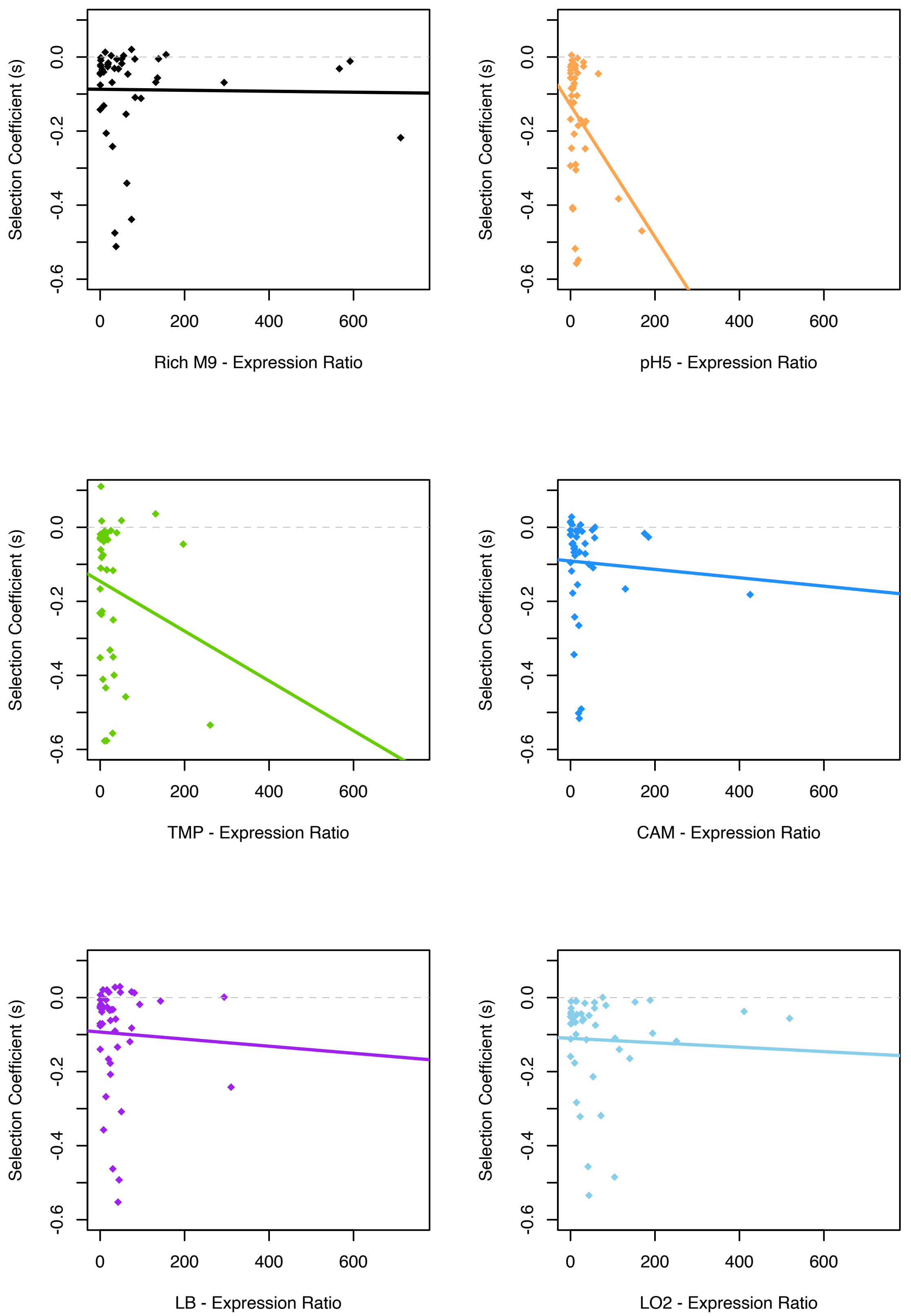

Supplementary Figure 2*. Selection coefficients of the transferred genes in six different environments plotted against the ratio of the gene expression level of the transferred ortholog to that of the endogenous copy, Supplementary data 4. M9: F1, 42 = 0.011, p = .918, r2 = -0.024; Ph5:* $F_{1,\,42}$ *= 5.484, p = .052,* r^2 *= 0.094; TMP:* $F_{1,\,42}$ *= 1.38, p = .246,* r^2 *= 0.009; CAM:* $F_{1,\,42}$ *= 0.152, p = .698,* r^2 *= -0.020; LB:* $F_{1,\,42}$ *= 0.0767, p = .783,* r^2 *= -0.021; LO2:* $F_{1,\,42}$ *= 0.841, p = .364,* r^2 *= -0.003.*

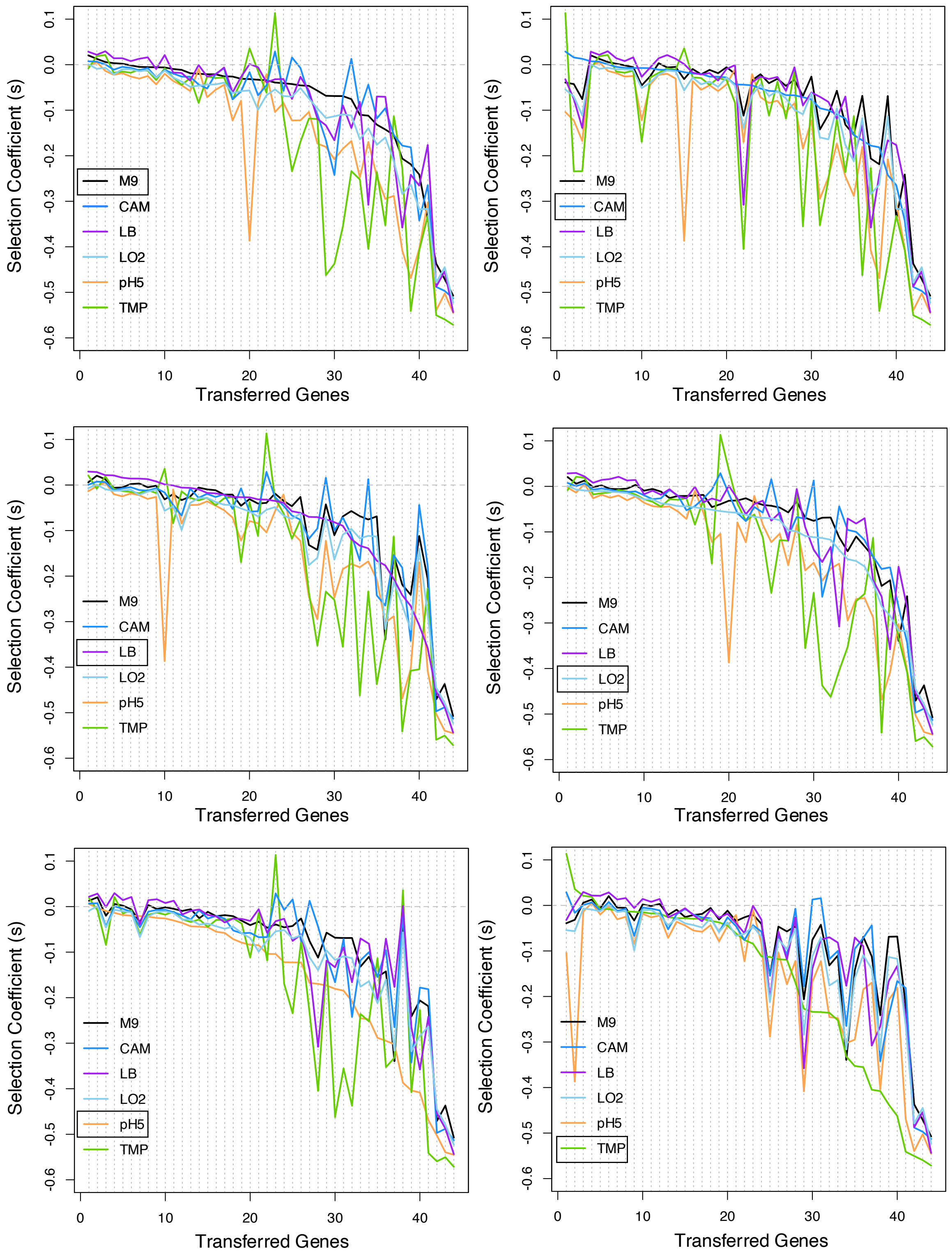

Supplementary Figure 3. *Selection coefficients of the transferred genes in six different environments. Transferred genes are ranked by their selection coefficient in the environment inside a black box. Colors indicate the six environments used in this study.*

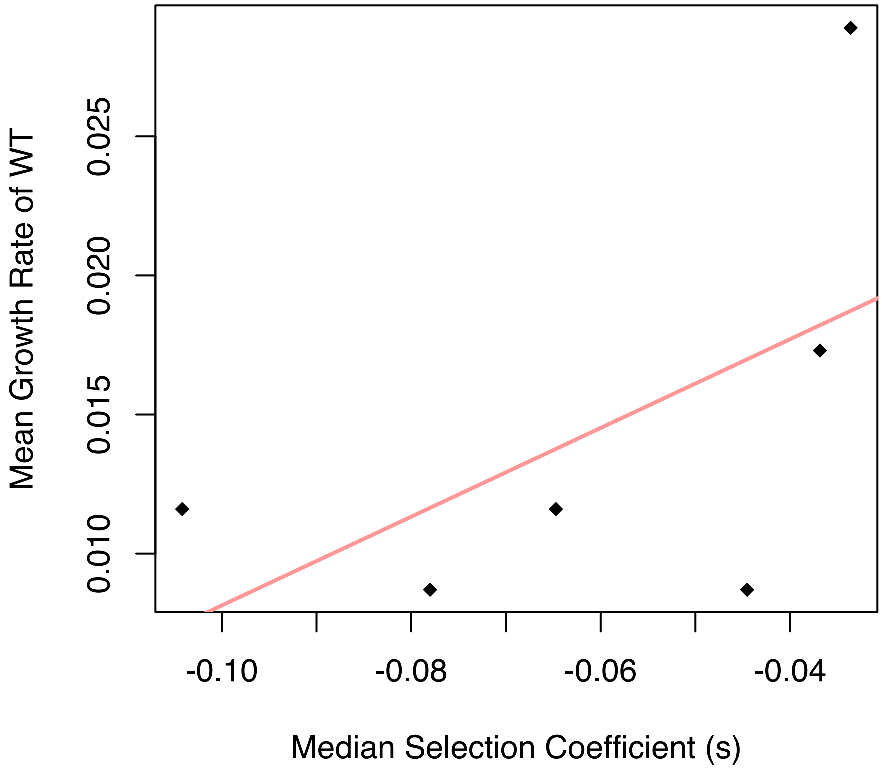

Supplementary Figure 4. *Mean growth rates of the wild type strain in each environment plotted against the median selection coefficient of the 44 transferred genes in each environment. ($F_{1, 4}$ = 1.887, p = .242, r^2 = 0.151)*
